# Quality of End-of-Life Care for People with Advanced Non-Small Cell Lung Cancer in Ontario: A Population-Based Study

**DOI:** 10.3390/curroncol28050286

**Published:** 2021-08-26

**Authors:** Catherine L. Goldie, Paul Nguyen, Andrew G. Robinson, Craig E. Goldie, Colleen E. Kircher, Timothy P. Hanna

**Affiliations:** 1 School of Nursing, Queen’s University, Kingston, ON K7L 3N6, Canada; 8cem14@queensu.ca; 2 ICES Queen’s, Queen’s University, Kingston, ON K7L 3L4, Canada; paul.nguyen@ices.on.ca (P.N.); tim.hanna@kingstonhsc.ca (T.P.H.); 3 School of Medicine, Queen’s University, Kingston, ON K7L 3N6, Canada; andrew.robinson@kingstonhsc.ca (A.G.R.); craig.goldie@kingstonhsc.ca (C.E.G.); 4 Division of Cancer Care and Epidemiology, Queen’s Cancer Research Institute, Kingston, ON K7L 3N6, Canada; 5 Department of Oncology, Queen’s University, Kingston, ON K7L 5P9, Canada

**Keywords:** lung cancer, end-of-life care, quality indicators, palliative care, health services research, Canada

## Abstract

Ensuring high quality end of life (EOL) care is necessary for people with advanced non-small-cell lung cancer (NSCLC), given its high incidence, mortality and symptom burden. Aggressive EOL care can adversely affect the quality of life of NSCLC patients without providing meaningful oncologic benefit. Objectives: (1) To describe EOL health services quality indicators and timing of palliative care consultation provided to patients dying of NSCLC. (2) To examine associations between aggressive and supportive care and patient, disease and treatment characteristics. Methods: This retrospective population-based cohort study describes those who died of NSCLC in Ontario, Canada from 2009–2017. Socio-demographic, patient, disease and treatment characteristics as well as EOL health service quality and use of palliative care consultation were investigated. Multivariable logistic regression models examined factors associated with receiving aggressive or supportive care. Results: Aggressive care quality indicators were present in 50.3% and supportive care indicators in 60.3% of the cohort (N = 37,203). Aggressive care indicators decreased between 2009 and 2017 (57.4% to 45.3%) and increased for supportive care (54.2% to 67.5%). Benchmarks were not met by 2017 in 3 of 4 cases. Male sex and greater comorbidity were associated with more aggressive EOL care and less supportive care. Older age was negatively associated and rurality positively associated with aggressive care. No palliative care consultation occurred in 56.0%. Conclusions: While improvements in the use of supportive rather than aggressive care were noted, established Canadian benchmarks were not met. Moreover, there is variation in EOL quality between groups and use of earlier palliative care must improve.

## 1. Introduction

In Canada, lung cancer is one of the most frequently diagnosed cancers and the leading cause of cancer-related death. It is estimated that 29,800 Canadians were diagnosed with lung cancer in 2020 and the 5-year net survival rate was 19% [1]. Lung cancer is also the second most commonly diagnosed cancer in Ontario with approximately 10,592 new cases in 2020 [2]. When lung cancer becomes metastatic, or stage IV, the median survival is less than 1 year [3]. Patients can experience symptoms specific to their disease process (such as chronic cough, dyspnea, or pain) or more generalized symptoms (such as fatigue, loss of appetite, or cachexia). These symptoms can lead to, or exacerbate, psychological symptoms (such as depression and anxiety). Patients with non-small-cell lung cancer (NSCLC) often present with advanced disease and a high symptom burden. These symptoms contribute to distress, suffering and diminished quality of life and require intensive resources. Given the incurable nature of metastatic, unresectable NSCLC, the goals of therapy should include an emphasis on patient-oriented outcomes which optimize quality of life and reduce unnecessarily aggressive end of life (EOL) care and increase supportive EOL care [4,5,6,7,8].

Measures of quality EOL cancer care are explicitly defined as measurable items of practice performance used to evaluate the quality of care provided by a healthcare organization [9]. Commonly used and previously identified aggressive EOL outcomes from administrative data include hospital-centric measures such as visits to the emergency department (ED) or admission to the hospital or intensive care unit (ICU) in last 30 days of life, receiving systemic therapy within the last 14 days of life, or death in hospital [10,11]. These correspond to high healthcare expenditures [12]. Supportive EOL care includes community care service measures such as physician home visits in the last two weeks of life, or nursing or personal support worker visits at home in the six months before death [10,11]. These supportive care services increase patient satisfaction with care while reducing the use of acute care services and lowering costs to the healthcare system [4,13]. Although there has been a general upward trend in the aggressiveness of cancer care offered near the EOL in Ontario and other jurisdictions [14,15], it is known that the timing of palliative care consultation can attenuate delivery of aggressive care [3,16,17,18,19].

Palliative care plays an important role in oncologic practice as it enhances quality of life at the EOL [10]. It comprises an “approach that improves the quality of life of patients and their families facing problems associated with life-threatening illness, through the prevention and relief of suffering by means of early identification and impeccable assessment and treatment of pain and other problems, physical, psychosocial and spiritual” [20]. Specialist palliative care teams focus on symptom assessment and control, advanced care planning and psychosocial support. Traditionally, palliative care consultation has been delivered late in the course of the disease, when it is evident that disease-modifying treatments are unsuccessful and patients are hospitalized with a high symptom burden [3,21]. Recent studies have suggested that palliative care services should be provided earlier in the disease trajectory, close to the diagnosis of incurable lung cancer, through outpatient settings to enhance quality of EOL care [22,23,24,25,26]. However, there is often insufficient funding for palliative care services which creates disparities in access [27,28,29]. Given the changing context of oncologic and palliative care, there is a need to continuously monitor trends in aggressive and supportive care offered to patients with advanced NSCLC to determine whether they are accessing resources to improve their quality of EOL and to inform the delivery of future healthcare services.

### Purpose

The purpose of this study is thus: (1) To describe EOL health service quality indicators and timing of palliative care consultation provided to patients who were diagnosed with NSCLC and died from cancer-related causes in Ontario, Canada, and (2) to examine associations between aggressive and supportive care by patient, disease and treatment characteristics.

## 2. Materials and Methods

### 2.1. Study Population

This was a population-based retrospective cohort study of patients diagnosed with NSCLC who died from cancer in Ontario, Canada from January 2009–December 2017. In Ontario (population 14.6 million in 2019), medical care is primarily provided under a single-payer universal health coverage. NSCLC diagnoses were identified before death date through the Ontario Cancer Registry (OCR) (starting from 1 January 1964) using the combination of specified ICD-O-3 morphology and topography codes (Table A1) for the bronchus and lung body site, which are held securely at ICES (formerly Institute for Clinical Evaluative Sciences). ICES is an independent, non-profit research institute which houses a comprehensive high-quality collection of health administrative claims and billing data in the province of Ontario, whose legal status under Ontario’s health information privacy law allows it to collect and analyze healthcare and demographic data, without consent, for health system evaluation and improvement. Patients with other non-NSCL cancer diagnoses, without a valid provincial health card and continuous coverage (5-year lookback) and without a cause of death due to cancer were excluded to minimize the risk of misclassifying treatment information. This study was approved by the Queen’s University Health Sciences Research Ethics Board (#6024258). This study followed the Strengthening the Reporting of Observational Studies in Epidemiology (STROBE) reporting guideline for cohort studies.

### 2.2. Data Sources

The OCR is a passive cancer registry that records every malignant neoplasm diagnosed in Ontario through a series of probabilistic linkages. The data involved in these linkages include death registry data, pathology reports, cancer center records and hospitalization records. Investigations have indicated a 98% capture rate for all cases of cancer diagnosed in the province of Ontario [30]. All information on all deaths, including the cause of death, registered in Ontario is provided by the Office of the Registrar General-Deaths (ORGD) data. Records of acute hospital inpatient and day surgery admissions and discharges, including transfers and deaths, were collected from the Canadian Institute for Health Information Discharge Abstract Database and Same Day Surgery (CIHI DAD and SDS) data. Emergency department visits were identified using the National Ambulatory Care Reporting System (NACRS). Records and assessments during the occupation of an adult inpatient mental health bed, including transfers and deaths, were collected by the Ontario Mental Health Reporting System (OMHRS). Records of physician consults or assessments in private offices, acute care and long-term care facilities; technical and professional components of diagnostic and therapeutic procedures; and surgical procedures were collected from the Ontario Health Insurance Plan (OHIP). All home care services provided or coordinated by Ontario’s Health Shared Services Ontario (HSSO), including government-funded home and community services, were identified using the Home Care Database (HCD). Records of radiation and systemic therapy were collected within Activity Level Reporting (ALR) data. Records of intravenous drugs approved for delivery in Ontario were documented in the New Drug Funding Program (NDFP) and drug benefits for all adults aged 65+ and those receiving social assistance were collected in the Ontario Drug Benefit (ODB) program. Chronic conditions (i.e., asthma, chronic obstructive pulmonary disease and congestive heart failure) were identified with ICES-derived datasets based on validated algorithms from chart abstraction. These data sets were linked using unique encoded identifiers and were analyzed at ICES.

### 2.3. Classification of Independent Variables

We stratified our study cohort of NSCLC patients who died of cancer into individuals who did and did not receive palliative intent anticancer treatment (including systemic therapy, radiotherapy or surgical metastasectomy). Though the primary achievement is in most cases palliation and disease control, it is acknowledged that in some cases life is also extended and this may be an important part of the treatment goal. This was accomplished using a combination of reported treatment intent in ALR and time-based rules to separate curative, adjuvant and palliative intent treatment in consultation with oncologists (T.H. and A.R.) and a palliative care physician (C.G.). For patients without any previous or concurrent treatment, palliative intent systemic therapy was identified using specific medications. Palliative intent radiotherapy was identified using the available intent of treatment or body site-dose fractionation course of treatment; palliative intent surgeries were identified based on surgical intervention codes related to resection of intracranial or liver tumours, or spinal cord compression. For patients treated with thoracic tumour resection surgery or curative concurrent chemoradiation (defined as overlapping treatment courses of systemic therapy with any intent and curative/adjuvant/neoadjuvant intent or palliative/unknown intent plus ≥16 fractions of radiotherapy), systemic therapy, radiotherapy and surgical interventions were considered palliative if any treatment occurred more than 14 weeks after surgery or more than 10 weeks after chemoradiation. Receipt of the first palliative anticancer therapy was selected as the estimated date of advanced incurable NSCLC diagnosis.

Socio-demographics, general health, disease and treatment characteristics, as well as characteristics of palliative care consultation, were described with death date. Socioeconomic status was based on neighborhood household income quintiles. The rurality of patient residence was characterized by the 2008 Rurality Index for Ontario [31]. The Elixhauser comorbidity score was used with 5-year lookback with the CIHI DAD [32]. Diagnostic codes for metastatic cancer and solid tumour without metastases were excluded.

The time from first cancer diagnosis in OCR to death and the best stage at first cancer diagnosis were measured. Complete information on second and subsequent NSCLC primaries were not available for the full study period. Lastly, time from first cancer diagnosis to death, to first palliative treatment and number of days between palliative care consultation and death were measured.

### 2.4. Classification of Dependent Variables

EOL health service quality indicators were primarily defined by previously established measures [10,11]. Measures of aggressive care included: (1) >1 ED visit, (2) >1 inpatient hospital admission, (3) ICU admission within 30 days of death, (4) systemic therapy within two weeks of death or (5) death in hospital. We also included an additional indicator of (6) mental health symptoms within 30 days of death identified by any hospitalization, ED and physician visits using DAD, OMHRS, NACRS and OHIP records using ICD-10 diagnosis for psychotic, nonpsychotic, substance use disorders and other social maladjustment problems. Measures of supportive care, based on prior measures, were: (1) palliative nursing or personal support worker home visit within 6 months of death or (2) physician home visit within two weeks of death. Denominators for end-of-life indicators differed depending on the eligibility criteria used to derive them. For example, if patients were hospitalized for more than two weeks before death, they would not be able to receive a physician or nursing home visit and were excluded from the percentage calculation of this indicator. The presence of one or more measure of aggressive or supportive care was calculated for each patient. The time between first palliative care consultation and death was determined using OHIP billing codes (A945/C945). These codes identify first palliative care consultation from a physician who spends a minimum of 50 min with them and/or their representative/family. This service includes a psychosocial assessment, comprehensive review of pharmacotherapy, appropriate counselling and consideration of appropriate community services. This coding definition of palliative care consultation was selected because it specifically captures the core activities required to comprehensively assess and respond to a patient’s palliative care needs.

### 2.5. Statistical Analysis

Socio-demographics, general health, disease and treatment characteristics, as well as timing of palliative care consultation, were summarized and EOL health service quality indicators were described among the whole cohort and those that did and did not receive palliative intent anticancer treatment. Pearson’s chi-squared tests were utilized for descriptive analysis. Multivariable logistic regression models examined factors associated with receiving aggressive or supportive care. As death in hospital was anticipated to be a major contributor to aggressive care, a sensitivity analysis was conducted by creating two composite scores for aggressive care: one including and one excluding the death in hospital indicator.

All variables independently associated with the quality EOL care at a significance level of 20% were added in the stepwise model selection. Temporal trends in outcomes by death year were also described and evaluated by the Cochran–Armitage test. All analyses were performed using the SAS Enterprise Guide 7.15.

## 3. Results

### 3.1. Cohort Description and Patient Demographics

**Figure 1:**
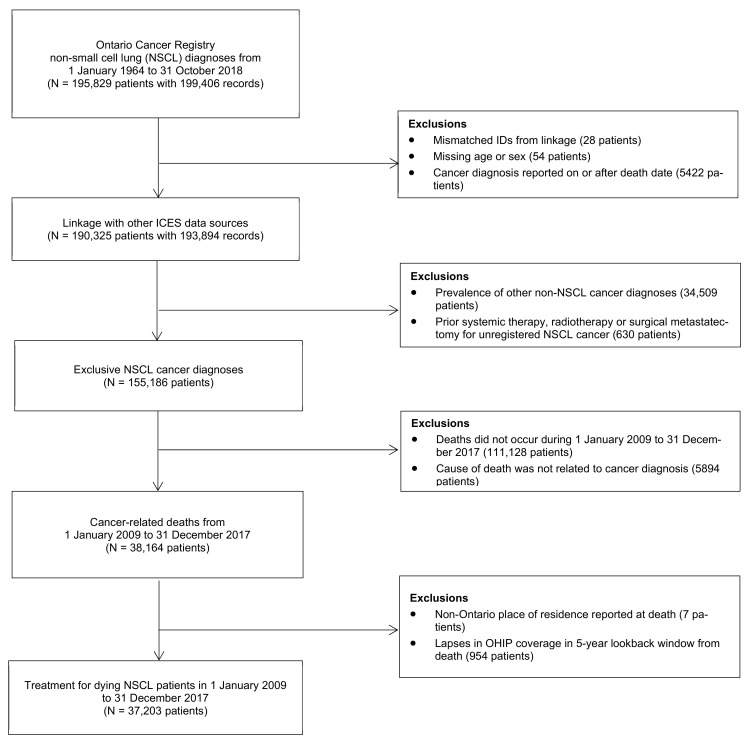
Identification of non-small cell lung cancer (NSCLC) patients who died of cancer-related causes in Ontario from 1 January 2009 to 31 December 2017.

In total, 37,203 patients meeting selection criteria were identified as dying from cancer-related causes between 1 January 2009 and 31 December 2017. Figure 1 provides information on identification of the study cohort.

The median age at death was 72 years; 60% were ≥70 years and the interquartile range (IQR) was 64–80 years (Table 1). There were greater proportions living in the lower income quintile neighborhoods, with 24.9% in the lowest quintile and 15.4% in the highest. A total of 62% lived in urban areas. Most patients (59.5%) had chronic obstructive pulmonary disease (COPD) and a mean Elixhauser comorbidity index of 2.0 (±1.9 standard deviation). Most patients in the sample were categorized as having stage IV cancer (54.9%) at first diagnosis and the median number of months between cancer diagnosis and death was 5. There were 47.1% (N = 17,535) of patients that did not receive any palliative intent chemotherapy, radiotherapy, or metastatic surgery treatment. Among those that did (N = 19,668), the mean time from first NSCLC diagnosis (any stage) to first palliative intent treatment was 8.9 (±26.9) months. Amongst the cohort, 56.0% did not receive any palliative care consultation, 18.1% received palliative care consultation within 30 days of death, 7.9% between 31–60 days before death, 10.8% between 61–180 before death and 7.3% more than 180 days before death.

### 3.2. End of Life Health Service Quality Indicators

**Figure 2:**
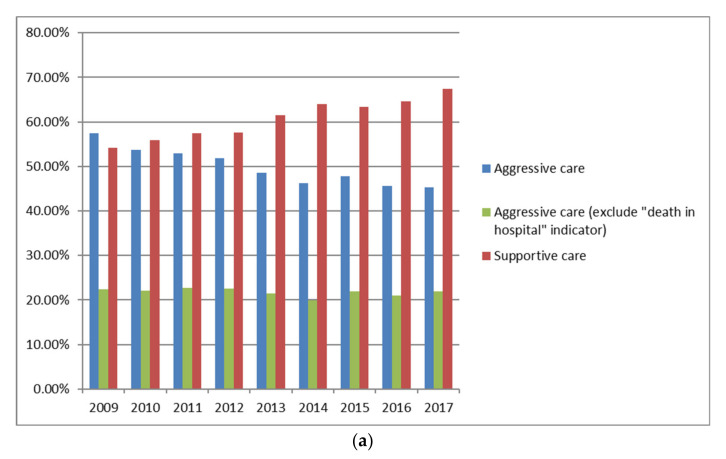

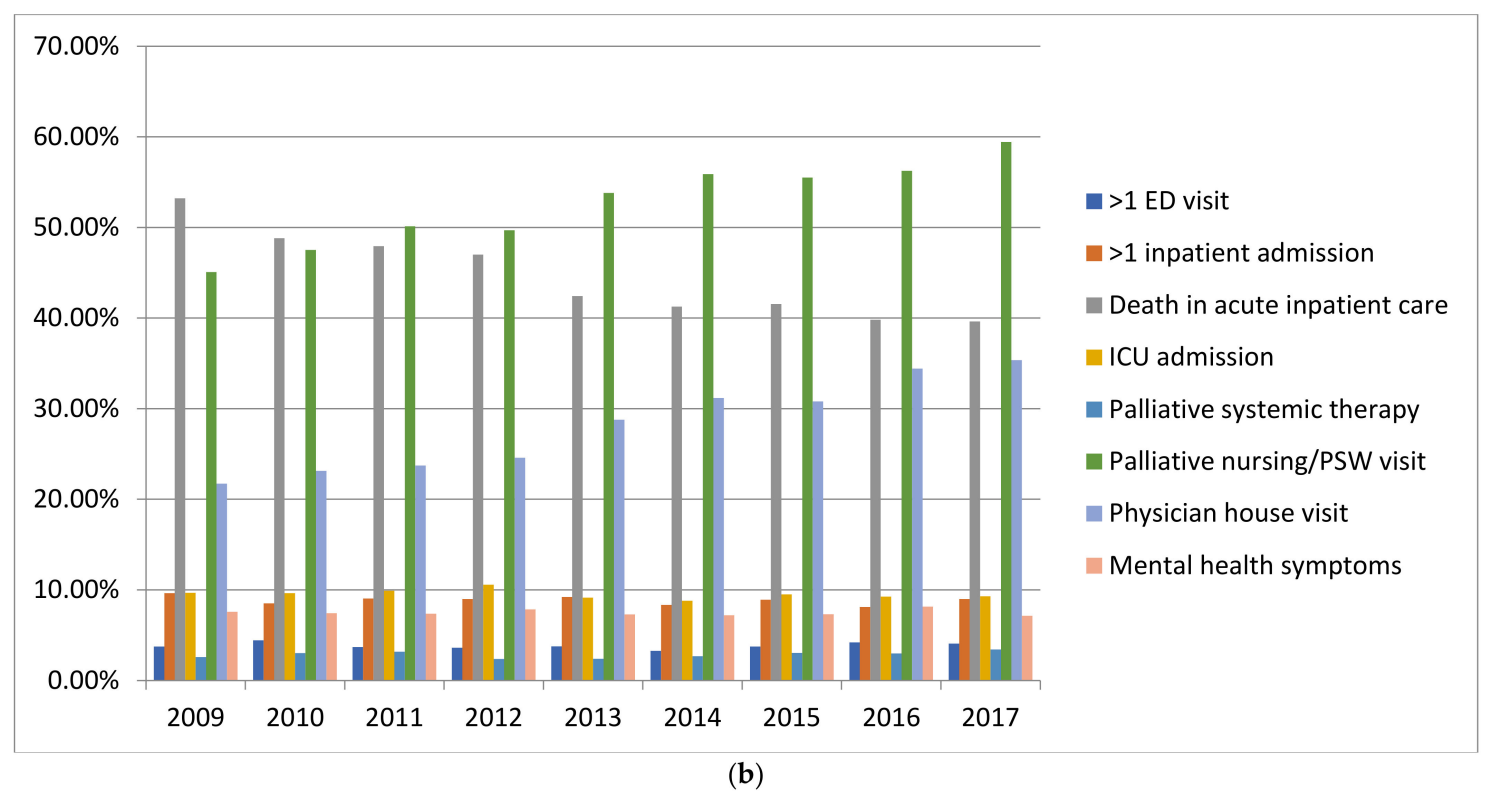
(**a**) Temporal trends for aggressive and supportive care offered to NSCLC patients who died from cancer-related causes from January 2009 to December 2017 (N = 37,203). (**b**) Temporal trends for end-of-life health service quality indicators for NSCL cancer patients who died from cancer-related causes from January 2009 to December 2017 (N = 37,203).

One or more indicators of aggressive care were present in 50.3% and for supportive care indicators were present in 60.3% of the cohort (N = 37,203) (Table 2). The number of patients who received aggressive care decreased over the study period (57.4% to 45.3%) while those receiving supportive care increased (54.2% to 67.5%) (all *p* < 0.001) (Figure 2a). The composite score for occurrence of any one or more of the aggressive care indicators, excluding death in hospital, was 21.8% and this remained stable across the study period. A total of 21% of patients who received palliative intent treatment had indicators of aggressive care (when excluding death in hospital) compared to 23.2% of patients who did not receive palliative intent treatment.

Of the 19,668 patients who received palliative intent anticancer therapy, there was an absolute proportional 8.0% decrease in aggressive care and a 5.0% increase in supportive care over the study period (all *p* < 0.001). Of the 17,535 patients who did not receive palliative intent anticancer treatment, there was a 14.5% decrease in aggressive care and an 18.5% increase in supportive care (all *p* < 0.001). The proportion of patients without anticancer treatment decreased from 52.5% in 2009 to 38.1% in 2017 (*p* < 0.001).

For individual EOL health service quality indicators, the most frequently occurring were palliative nursing or personal support worker home visit within 6 months of death (52.2%), death in hospital (45.0%) and physician home visit within two weeks of death (27.8%). The percentage of palliative nursing or personal support worker home visits and physician home visits increased from 2009–2017, death in hospital decreased, and ED visits, inpatient hospital admissions, ICU admission, systemic therapy within two weeks of death and mental health symptoms within 30 days of death were relatively stable (Figure 2b). Mental health symptoms were documented in 7.5% of the cohort.

**Figure 3:**
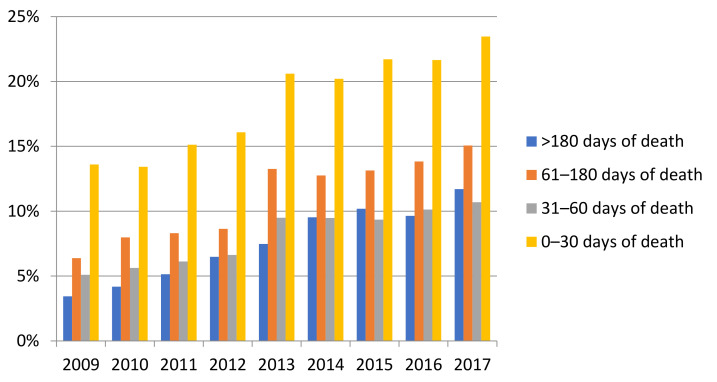
Temporal trends for palliative care consultation of NSCL cancer patients who died with cancer-related causes from January 2009 to December 2017 (N = 37,203).

The receipt of aggressive and supportive care varied between patients who did and did not receive palliative intent anti-cancer treatment. Among those that had palliative intent treatment (N = 19,668), 45.6% received aggressive care and 70.3% received supportive care whereas 55.5% of patients who did not receive palliative intent treatment (N = 17,535) received aggressive care and 48.1% received supportive care. Amongst the full cohort, the percentage of patients who received palliative care consultation before death increased for all time categories (>180, 61–180, 31–60, ≤30 days) between 2009 and 2017 (Figure 3).

When examining EOL health service quality indicators according to patient, disease and treatment characteristics, we found that patients who received aggressive care were slightly younger (70.9 versus 72.7 years), were more likely to be male (odds ratio (OR) = 1.21 (95% confidence interval (CI) 1.16–1.26)), less likely to live in urban (OR = 0.59 (95% CI 0.53–0.66)) or suburban (OR = 0.73 (95% CI 0.66–0.81)) environments compared with rural areas, more likely to have chronic obstructive pulmonary disease (OR = 1.05 (95% CI 1.00–1.10)), congestive heart failure (OR = 1.12 (95% CI 1.05–1.19)) or 2–3 (OR 1.46 (95% CI 1.39–1.54)) or 4+ (OR 1.66 (95% CI 1.55–1.77)) comorbid conditions than patients who did not receive aggressive care (Table 3). Age differences may explain discrepancies in medical care received by cancer patients near death with older patients preferring life-prolonging therapies less likely to receive them than younger patients [33]

While patients who received aggressive care were more likely to have prior adjuvant chemotherapy or curative concurrent chemoradiation (OR = 1.18 (95% CI 1.08–1.28)), they were less likely to have palliative intent anticancer therapy (OR= 0.87 (95% CI 0.83–0.92)). Finally, patients who had a palliative care consultation earlier were less likely to have aggressive care (e.g., 61–180 days OR = 0.49 (0.46–0.53)). A sensitivity analysis revealed that results were similar when the death in hospital indicator was excluded from the composite score for aggressive care (data not shown). However, less regional variation in aggressive care was observed in the regression analysis after removing death in hospital and there was a greater effect of ‘earlier’ palliative care on reducing aggressive care and slightly less impact of greater time from first cancer diagnosis to death on reducing aggressive care.

Patients who received supportive care were less likely to be men (OR = 0.95 (95% CI 0.91–1.00)), live in an urban versus rural setting (OR = 0.82 (95% CI 0.75–0.89)), have congestive heart failure (OR = 0.88 (95% CI 0.82–0.94)), have had prior adjuvant therapy or curative concurrent chemoradiation (OR = 0.83 (95% CI 0.76–0.92)). They had fewer comorbidities and were more likely to have lived in higher income neighborhoods, to have received palliative intent anticancer therapy (OR = 1.49 (95% CI 1.41–1.57)) and access to palliative care consultation before death than patients who did not receive supportive care.

## 4. Discussion

This population-based study of more than 37,000 individuals who died from NSCLC between 2009 and 2017 demonstrates that patients with advanced NSCLC continue to be heavy users of the healthcare system at the EOL. Four specific points are worth emphasizing. First, the number of patients who received aggressive care decreased considerably over the study period (2009–2017) while patients receiving supportive care increased substantially. This is encouraging as it indicates a positive shift in the quality of EOL care offered to patients with NSCLC. This shift is likely the result of better symptom control, changing practice patterns and the emergence of seminal research demonstrating the efficacy of early palliative care on patient-reported quality of EOL care [3].

Second, Barbera et al. [10] established Canadian benchmarks for EOL quality indicators using 33 healthcare service regions across British Columbia, Alberta, Ontario and Nova Scotia using data from 200,025 cancer deaths. They documented considerable variation in quality indicators across provinces but determined the following benchmark rates using funnel plots to graph each region’s age- and sex-adjusted indicator rates. Benchmark rates from top-performing regions were: single emergency department visit, 34% (our analysis used >1 ER visit); intensive care unit admission, 2%; physician home visit, 34%; home care visits, 63%; and death in hospital, 38%. For all indicators, our cohort did not attain the proposed benchmarks, with only one benchmark reached among the subgroup dying in 2017 (physician home visits).

When comparing individual aggressive care indicators to findings from an international comparative study of lung cancer deaths occurring in 2010 among adults >65 years in developed countries [12], we found that the percentage of patients (N = 4467 decedents) who were admitted to an ICU within 30 days of death were similar (9.56% versus 8.5%) to previous Canadian estimates while our findings documented a lower rate of death in hospital (45.0% versus 54.1%). When considered alongside international data from broadly similar health systems examining EOL quality indicators, patients with metastatic NSCLC did not receive treatment as ideally recommended by the benchmarks [34,35,36]. For instance, a retrospective cohort study of Australian patients with metastatic NSCLC (N = 6041) conducted between 2003–2010 found that only 5% of the cohort received intensive care and <1% chemotherapy at the EOL while a much larger proportion of patients died in acute hospitals (42%) and had a length of stay of greater than 14 days in the last month of life (61%) [35]. Another study examining lung cancer patients (N = 79,746) who died (between 2010–2011 or between 2015–2016) within a French national hospital database demonstrated a higher rate of aggressive care indicators during the last 30 days of life than was demonstrated in this study [36].

Our study also examined mental health symptoms within 30 days of death. This novel measure was examined because mental health symptoms are frequently underdiagnosed among people dying from advanced cancer and are a significant contributor to suffering, symptom burden and poorer quality of life than patients without these symptoms [37,38,39]. Prevalence estimates (7.49%) for mental health symptoms in this cohort were lower than have been previously described in longitudinal studies of patients with lung cancer [40,41]. This is likely attributable to differences across treatment settings, tools used to collect information about mental health symptoms and selected research designs.

Compared to a previous report of aggressive care offered to a smaller sample of patients (N = 5855) who died of lung cancer in Ontario (using similar administrative data holdings from ICES in 2002), our findings showed a reduction in the percentage of patients who died in hospital (45.0% versus 59.5%) and received systemic therapy (2.8% versus 4.6%) administered within the last two weeks of life while we noted an increase in ICU admission (9.6% versus 5.5%) in the last two weeks of life [10]. It is not clear why the percentage of patients who were admitted to ICU increased from previous reports and thus further study is warranted. This might be the result of a global increase in ICU capacity across Ontario resulting from expanded billing codes for non-vented patients; conversion of ‘step down’ units to ICUs or increased admission/discharge rates. This finding could also represent an attitude shift in short stay admissions to ICU or optimism surrounding the emergence of new immunotherapies that are associated with significant autoimmune toxicities that may require critical care admission and management [42,43,44].

Third, our study provides unique insights into socio-demographics, general health, disease and treatment characteristics that impact access to EOL quality indicators. We found that male sex and increased comorbidity were associated with more aggressive EOL care and less supportive care. These clinically and statistically significant differences persisted in adjusted analyses. This sex-based finding is consistent with past research demonstrating that men with cancer receive more aggressive EOL care than women [26,36,45,46]. Possible explanations for these results include the existence of sex differences in the content of patient–physician discussions about EOL care, the way that patients use the information from these discussions and differential communication and treatment preferences [45]. We also found that patients who received aggressive care were more likely to have prior adjuvant systemic therapy or concurrent chemoradiation. There are several potential reasons for this: misclassification, patient, or treatment factors. For example, if a patient had a rapid recurrence and received systemic therapy within the 14-week time-period, they would be misclassified as having adjuvant therapy when its actual intent was palliative; this situation would also indicate more aggressive disease. Alternatively, patterns in patient preferences might exist; those who elect to undergo adjuvant systemic therapy might also be more likely to pursue aggressive EOL care. This finding might also represent death from complications of adjuvant systemic therapy and/or concurrent therapy.

Finally, we found that 56% of the cohort did not receive palliative care consultation before death. This is problematic as it is known that patients receiving palliative care consultation have better symptom control, death preparation, less aggressive care and overall quality of death [47,48]. It is encouraging to note that access to palliative care consultation before death increased in all time categories over the study period; moreover, the proportion of early palliative care consultation (defined as >60 days before death) increased from 34% of all consultations for patients dying in 2009, to 44% in 2017 [21]. Timing of referral to palliative care consultation for patients may be influenced by patient preference or lack of availability of palliative care providers and consensus of when referral is most appropriate. We found that receipt of palliative care consultation one month or more before death was associated with less aggressive care compared to patients who did not receive a palliative care consult. This finding is congruent with previous literature [48,49].

Strengths of our study include its use of real-world population-based data. Building an understanding of population-level characteristics of quality EOL care is especially important given the changing context of oncologic and palliative care and associated resource constraints inherent to Ontario’s publicly funded system. For example, there has been a shift toward earlier palliative care consultation and the use of an array of more effective and sometimes more toxic systemic therapies, all of which could alter the EOL experience for people with incurable NSCLC. The linked administrative data sources available from hospitals across the province of Ontario provided perspectives on the whole population of NSCLC patients treated between 2009 and 2017, avoiding selection biases inherent to other research designs.

### Limitations

However, our study has important limitations. We did not directly measure quality of life, only measures of health utilization that might correlate with quality of life. We also did not examine the setting in which palliative care consultation was offered and did not evaluate quality of care earlier in the trajectory of palliative care (e.g., after patients we diagnosed as incurable/palliative but before they entered the last few months of life). The Canadian benchmarks for EOL quality indicators we compared our results to were not specifically developed for lung cancer. Furthermore, benchmark measures are metrics used to gauge the quality and performance of a health system but do not imply that care delivered to an individual was inappropriate. For instance, there are circumstances where being admitted to ICU at the EOL or dying in hospital is appropriate clinical care. Canadian EOL quality benchmarks should continue to evolve to reflect patient preferences as well as changes to the structure and function of the healthcare system.

## 5. Conclusions

This work has significant clinical, resource allocation and policy implications for the treatment of patients with advanced NSCLC. While improvements in the use of supportive rather than aggressive care were noted, established benchmarks were not met by the cohort. There are variations in EOL quality between groups, some of which may be inappropriate and need to be addressed to improve the equity of care. Moreover, we need to make gains in use of earlier palliative care. To optimize the quality of EOL care, we should continuously monitor EOL health service quality indicators against established Canadian benchmarks. Future research must continue to investigate sex-based differences and patient-oriented outcomes to optimize quality of EOL care practices for patients with NSCLC.

## Figures and Tables

**Table 1:** Patient socio-demographics, general health, disease and treatment characteristics.

	All Patients (N = 37,203)		Palliative Treatment (Systemic Therapy, Radiotherapy, Metastasis Surgery) (N = 19,668)		Non-Palliative Treatment (N = 17,535)	
Characteristics	Frequency	Percent (%)	Frequency	Percent (%)	Frequency	Percent (%)
Socio-demographics						
Age						
20–49	853	2.29	660	3.36	193	1.10
50–59	4382	11.78	3139	15.96	1243	7.09
60–69	9773	26.27	6299	32.03	3474	19.81
70–79	12,337	33.16	6480	32.95	5857	33.40
80+	9858	26.50	3090	15.71	6768	38.60
Sex						
Male	19,701	52.96	10,408	52.92	9293	53.00
Female	17,502	47.04	9260	47.08	8242	47.00
Neighborhood income quintile at death date						
Missing	121	0.33	52	0.26	69	0.39
1 (Lowest)	9277	24.94	4625	23.52	4652	26.53
2	8306	22.33	4360	22.17	3946	22.50
3	7286	19.58	3921	19.94	3365	19.19
4	6485	17.43	3540	18.00	2945	16.79
5 (Highest)	5728	15.40	3170	16.12	2558	14.59
Place of residence (LHIN) at death date						
Erie St. Clair	2329	6.26	1216	6.18	1113	6.35
South West	3021	8.12	1517	7.71	1504	8.58
Waterloo Wellington	1912	5.14	1019	5.18	893	5.09
Hamilton Niagara Halimand Brant	4902	13.18	2363	12.01	2539	14.48
Central West	1356	3.64	770	3.91	586	3.34
Mississauga Halton	2081	5.59	1157	5.88	924	5.27
Toronto Central	2547	6.85	1406	7.15	1141	6.51
Central	3251	8.74	1857	9.44	1394	7.95
Central East	4563	12.27	2341	11.90	2222	12.67
South East	2219	5.96	1220	6.20	999	5.70
Champlain	3764	10.12	2138	10.87	1626	9.27
North Simcoe Muskoka	1669	4.49	785	3.99	884	5.04
North East	2711	7.29	1468	7.46	1243	7.09
North West	878	2.36	411	2.09	467	2.66
Rurality status at death date						
Missing	26	0.07	10	0.05	16	0.09
Yes	5850	15.72	2906	14.78	2944	16.79
No	31,327	84.21	16,752	85.17	14,575	83.12
Type of environment at death date						
NA/Missing	429	1.15	208	1.06	221	1.26
Urban (RIO < 10)	22,948	61.68	12,543	63.77	10,405	59.34
Suburban (10 ≤ RIO < 40)	9531	25.62	4839	24.60	4692	26.76
Rural (40 ≤ RIO)	4295	11.54	2078	10.57	2217	12.64
General health characteristics						
Chronic comorbidities prior to death						
Asthma	6445	17.32	3172	16.13	3273	18.67
Chronic obstructive pulmonary disease	22,152	59.54	10,801	54.92	11,351	64.73
Congestive heart failure	6627	17.81	2482	12.62	4145	23.64
Elixhauser comorbidity index *						
0–1	18,373	49.39	10,776	54.79	7597	43.32
2–3	11,854	31.86	6000	30.51	5854	33.38
4+	6976	18.75	2892	14.70	4084	23.29
Disease and treatment characteristics						
Best stage of first cancer diagnosis						
Missing	4909	13.20	962	4.89	3947	22.51
0/I	2866	7.70	1153	5.86	1713	9.77
II	1877	5.05	933	4.74	944	5.38
III	7090	19.06	4065	20.67	3025	17.25
IV	20,405	54.85	12,517	63.64	7888	44.98
Multiple primaries	56	0.15	38	0.19	18	0.10
Number of months from first cancer diagnosis to death						
0–<1	5692	15.30	582	2.96	5110	29.14
1–<2	4382	11.78	1765	8.97	2617	14.92
2–<3	3245	8.72	1783	9.07	1462	8.34
3–<6	5769	15.51	3711	18.87	2058	11.74
6–<12	5854	15.74	4053	20.61	1801	10.27
12+	12,261	32.96	7774	39.53	4487	25.59
Number of months from first cancer diagnosis to palliative intent treatment						
No treatment	17,535	47.13	0	0.00	17,535	100
0–<1	6777	18.22	6777	34.46	0	0.00
1–<2	4809	12.93	4809	24.45	0	0.00
2–<3	1819	4.89	1819	9.25	0	0.00
3–<6	1601	4.30	1601	8.14	0	0.00
6–<12	1460	3.92	1460	7.42	0	0.00
12+	3202	8.61	3202	16.28	0	0.00
Prior adjuvant systemic therapy or concurrent chemoradiation						
Prior to death date	2845	7.65	1833	9.32	1012	5.77

RIO, Rurality Index for Ontario; LHIN, Local Health Integration Network; * Five-year lookback from death: Total comorbidity score excludes indices for metastatic cancer and solid tumour without metastases.

**Table 2:** End of life (EOL) health service quality indicators for all patients and by receipt of palliative intent treatment.

	All Patients N = 37,203		Palliative Intent Treatment N = 19,668		No Palliative Intent Treatment N = 17,535		*p*-Value
Outcome	Frequency	Percent (%)	Frequency	Percent (%)	Frequency	Percent (%)	
Aggressive care *	18,692	50.26	8962	45.58	9730	55.51	<0.001
Aggressive care £	7833	21.81	3937	20.59	3896	23.20	<0.001
Supportive care **	20,515	60.30	13,139	70.30	7376	48.11	<0.001
Individual indicators of aggressive care							
>1 Emergency department visit ****	1372	3.84	773	4.05	599	3.59	0.025
>1 Hospital inpatient admission ****	3174	8.88	1524	7.99	1650	9.90	<0.001
ICU admission ****	3556	9.56	1311	6.67	2245	12.80	<0.001
Death in hospital	16,756	45.04	7731	39.31	9025	51.47	<0.001
Palliative systemic therapy ***	1056	2.84	1056	5.37	0	0.00	<0.001
Individual indicators of supportive care							
Palliative nursing or PSW home visit *****	19,385	52.15	12,562	63.89	6823	38.97	<0.001
Physician home visit ***	9070	27.82	5686	32.05	3384	22.77	<0.001
Other							
Mental health symptoms ****	2786	7.49	1427	7.26	1359	7.75	0.070
Number of days from first access to palliative care consultation ¥ to death							
>180	2703	7.27	2086	10.61	617	3.52	<0.001
61–180	4000	10.75	2897	14.73	1103	6.29	
31–60	2930	7.88	1975	10.04	955	5.45	
≤30	6725	18.08	3481	17.70	3244	18.50	
None	20,845	56.03	9229	46.92	11,616	66.24	

PSW, Personal Support Worker; * Composite score for occurrence of any one or more of the aggressive care indicators; ** Composite score for occurrence of any one or more of the supportive care indicators; *** Within 2 weeks of death; **** Within 30 days of death; ***** Within 6 months of death; Notes: Denominators for end-of-life indicators differed depending on the eligibility criteria used to derive them; Denominator for physician home visits excludes patients with ≥2-week hospital stay before death; Denominators for emergency department visits and inpatient admissions exclude patients with ≥30-day hospital stay before death; Denominator for palliative nursing or PSW home visit excludes patients with ≥6-month hospital stay before death; Denominators for composite scores for aggressive and supportive care are different due to the patients excluded from their respective individual indicators. £ Composite score for occurrence of any one or more of the aggressive care indicators, except for death in hospital. ¥ OHIP billing codes: A945, C945.

**Table 3:** Multivariable logistic regression analyses for end of life (EOL) health service quality indicators with socio-demographics, general health, disease and treatment characteristics of NSCL cancer patients who died from cancer-related causes from January 2009 to December 2017 (N = 37,203).

Characteristics	Aggressive Care			Supportive Care		
	Yes	No	Adjusted Stepwise Model	Yes	No	Adjusted Stepwise Model
	N = 18,692 (%)	N = 18,499 (%)	Odds Ratio (95% CI)	N = 20,515 (%)	N = 13,507 (%)	Odds Ratio (95% CI)
Socio-demographics						
Death year *						
Mean ± SD	2012.56 ± 2.56	2012.95 ± 2.54	0.96 (0.95–0.97)	2012.97 ± 2.55	2012.53 ± 2.53	1.03 (1.02–1.04)
Age at death date						
20–49	523 (2.80)	330 (1.78)	Reference	514 (2.51)	274 (2.03)	
50–59	2392 (12.80)	1990 (10.76)	0.69 (0.59–0.81)	2695 (13.14)	1382 (10.23)	
60–69	5192 (27.78)	4576 (24.74)	0.56 (0.48–0.66)	5656 (27.57)	3366 (24.92)	
70–79	6217 (33.26)	6114 (33.05)	0.45 (0.39–0.53)	6797 (33.13)	4408 (32.63)	
80+	4368 (23.37)	5489 (29.67)	0.31 (0.27–0.36)	4853 (23.66)	4077 (30.18)	
Sex						
Female	8218 (43.97)	9277 (50.15)	Reference	9961 (48.55)	6164 (45.64)	Reference
Male	10,474 (56.03)	9222 (49.85)	**1.21 (1.16–1.26)**	10,554 (51.45)	7343 (54.36)	**0.95 (0.91–1.00)**
Neighborhood income quintile at death date						
1 Lowest	4747 (25.40)	4528 (24.48)		4820 (23.50)	3645 (26.99)	Reference
2	4227 (22.61)	4079 (22.05)		4583 (22.34)	3007 (22.26)	1.13 (1.06–1.22)
3	3658 (19.57)	3623 (19.58)		4105 (20.01)	2580 (19.10)	1.15 (1.07–1.23)
4	3214 (17.19)	3268 (17.67)		3673 (17.90)	2246 (16.63)	1.19 (1.11–1.29)
5 Highest	2781 (14.88)	2945 (15.92)		3284 (16.01)	1973 (14.61)	1.18 (1.09–1.27)
Place of residence at death date						
Erie St. Clair	1012 (5.41)	1316 (7.11)	0.60 (0.53–0.69)	1469 (7.16)	732 (5.42)	2.09 (1.81–2.41)
South West	1660 (8.88)	1360 (7.35)	0.97 (0.86–1.09)	1605 (7.82)	1124 (8.32)	1.41 (1.24–1.61)
Waterloo Wellington	784 (4.19)	1128 (6.10)	0.71 (0.62–0.80)	1372 (6.69)	465 (3.44)	2.86 (2.47–3.32)
Hamilton Niagara Halimand Brant	2105 (11.26)	2797 (15.12)	0.68 (0.61–0.76)	2870 (13.99)	1698 (12.57)	1.92 (1.71–2.15)
Central West	824 (4.41)	532 (2.88)	1.57 (1.36–1.80)	697 (3.40)	492 (3.64)	1.35 (1.15–1.58)
Mississauga Halton	1045 (5.59)	1036 (5.60)	1.15 (1.01–1.29)	1213 (5.91)	675 (5.00)	1.68 (1.46–1.93)
Toronto Central	1210 (6.47)	1337 (7.23)	Reference	1251 (6.10)	1040 (7.70)	Reference
Central	1591 (8.51)	1660 (8.97)	1.09 (0.98–1.22)	1577 (7.69)	1325 (9.81)	0.87 (0.77–0.98)
Central East	2709 (14.49)	1854 (10.02)	1.46 (1.31–1.62)	2326 (11.34)	1778 (13.16)	1.11 (0.99–1.25)
South East	1143 (6.11)	1075 (5.81)	0.82 (0.72–0.94)	980 (4.78)	1074 (7.95)	0.88 (0.77–1.01)
Champlain	1714 (9.17)	2047 (11.07)	0.70 (0.63–0.78)	2237 (10.90)	1268 (9.39)	1.78 (1.57–2.01)
North Simcoe Muskoka	830 (4.44)	839 (4.54)	0.73 (0.64–0.84)	1036 (5.05)	535 (3.96)	2.13 (1.82–2.48)
North East	1599 (8.55)	1107 (5.98)	1.02 (0.90–1.15)	1539 (7.50)	879 (6.51)	1.99 (1.73–2.28)
North West	466 (2.49)	411 (2.22)	0.91 (0.76–1.08)	343 (1.67)	422 (3.12)	0.66 (0.55–0.80)
Rurality status at death date						
Yes	3524 (18.85)	2322 (12.55)	1.09 (1.00–1.20)	3089 (15.06)	2166 (16.04)	0.94 (0.88–1.00)
Type of environment at death date						
Urban (RIO < 10)	10,737 (57.44)	12,209 (66.00)	**0.59 (0.53–0.66)**	12,620 (61.52)	8397 (62.17)	**0.82 (0.75–0.89)**
Suburban (10 ≤ RIO < 40)	4990 (26.70)	4536 (24.52)	**0.73 (0.66–0.81)**	5434 (26.49)	3373 (24.97)	1.00 (0.92–1.10)
Rural (40 ≤ RIO)	2711 (14.50)	1580 (8.54)	Reference	2250 (10.97)	1563 (11.57)	Reference
**General health characteristics**						
Chronic comorbidities prior to death						
Asthma	3283 (17.56)	3156 (17.06)		3464 (16.89)	2399 (17.76)	
Chronic obstructive pulmonary disease	11,397 (60.97)	10,743 (58.07)	**1.05 (1.00–1.10)**	11,889 (57.95)	8326 (61.64)	0.96 (0.91–1.01)
Congestive heart failure	3643 (19.49)	2984 (16.13)	**1.12 (1.05–1.19)**	3084 (15.03)	2795 (20.69)	**0.88 (0.82–0.94)**
Elixhauser comorbidity index **						
0–1	8389 (44.88)	9982 (53.96)	Reference	11,086 (54.04)	6106 (45.21)	Reference
2–3	6371 (34.08)	5478 (29.61)	**1.46 (1.39–1.54)**	6149 (29.97)	4547 (33.66)	0.76 (0.72–0.81)
4+	3932 (21.04)	3039 (16.43)	**1.66 (1.55–1.77)**	3280 (15.99)	2854 (21.13)	0.68 (0.64–0.74)
**Disease and treatment characteristics**						
Number of months from first cancer diagnosis to death						
0–<1	4100 (21.93)	1592 (8.61)	Reference	1233 (6.01)	3618 (26.79)	Reference
1–<2	2216 (11.86)	2164 (11.70)	0.41 (0.37–0.45)	1988 (9.69)	1824 (13.50)	2.59 (2.35–2.85)
2–<3	1512 (8.09)	1731 (9.36)	0.39 (0.35–0.42)	1878 (9.15)	1068 (7.91)	3.69 (3.31–4.10)
3–<6	2617 (14.00)	3150 (17.03)	0.38 (0.35–0.42)	3754 (18.30)	1638 (12.13)	4.37 (3.97–4.81)
6–<12	2700 (14.44)	3153 (17.04)	0.39 (0.36–0.43)	3977 (19.39)	1556 (11.52)	4.75 (4.31–5.23)
12+	5547 (29.68)	6709 (36.27)	0.37 (0.35–0.41)	7685 (37.46)	3803 (28.16)	4.04 (3.71–4.41)
Prior adjuvant systemic therapy for thoracic tumour resection or curative concurrent chemoradiation						
Prior to death date	1463 (7.83)	1382 (7.47)	**1.18 (1.08–1.28)**	1752 (8.54)	905 (6.70)	**0.83 (0.76–0.92)**
Palliative intent systemic therapy, radiotherapy and metastasis surgery						
Prior to death date	8962 (47.95)	10,700 (57.84)	**0.87 (0.83–0.92)**	13,139 (64.05)	5551 (41.10)	**1.49 (1.41–1.57)**
Number of days from first access to palliative care consultation *** to death						
>180	983 (5.26)	1719 (9.29)	**0.56 (0.51–0.62)**	2165 (10.55)	467 (3.46)	3.46 (3.09–3.88)
61–180	1334 (7.14)	2665 (14.41)	**0.49 (0.46–0.53)**	3189 (15.54)	674 (4.99)	3.41 (3.10–3.76)
31–60	1024 (5.48)	1906 (10.30)	**0.53 (0.49–0.58)**	2121 (10.34)	625 (4.63)	2.75 (2.49–3.04)
≤30	3764 (20.14)	2959 (16.00)	1.06 (0.99–1.12)	3454 (16.84)	2510 (18.58)	1.55 (1.45–1.66)
None	11,587 (61.99)	9250 (50.00)	Reference	9586 (46.73)	9231 (68.34)	Reference

CI, Confidence Interval; RIO, Rurality Index for Ontario; * Odds ratio measured as per 1-year increase; ** Five-year lookback from death: Total comorbidity score excludes indices for metastatic cancer and solid tumour without metastases; *** OHIP billing codes: A945, C945; Notes: Aggressive and supportive care outcomes were both composite scores for occurrence of any one or more the respective indicators; In the adjusted stepwise model, variables with a significance level of 0.20 were used for model selection and variables with a significance level of 0.10 remained in the final model. The stepwise model selection approach excluded variables that do not significantly contribute to the final model; Missing values for the outcomes and variables were first excluded before running the logistic regression analyses. Significant associations discussed in the text are bolded.

## Data Availability

The dataset from this study is held securely in coded form at ICES. While legal data sharing agreements between ICES and data providers (e.g., healthcare organizations and government) prohibit ICES from making the dataset publicly available, access may be granted to those who meet pre-specified criteria for confidential access, available at www.ices.on.ca/DAS, (accessed on 24 August 2021 (email: das@ices.on.ca)). The full dataset creation plan and underlying analytic code are available from the authors upon request, understanding that the computer programs may rely upon coding templates or macros that are unique to ICES and are therefore either inaccessible or may require modification.

## Appendix A

**Table A1:** Morphology and topography codes for identifying non-small cell lung cancer.

ICD-O-3	Term
8000/3	Neoplasm, malignant
8001/3	Tumour cells, malignant
8004/3	Malignant tumour, fusiform cell type
8010/3	Carcinoma NOS
8012/3	Large cell carcinoma NOS
8020/3	Carcinoma, undifferentiated NOS
8021/3	Carcinoma, anaplastic NOS
8022/3	Pleomorphic carcinoma
8030/3	Giant cell and spindle cell carcinoma
8031/3	Giant cell carcinoma
8032/3	Spindle cell carcinoma
8034/3	Polygonal cell carcinoma
8050/3	Papillary carcinoma NOS
8051/3	Verrucous carcinoma NOS
8052/3	Papillary squamous cell carcinoma
8070/3	Squamous cell carcinoma NOS
8070/6	Squamous cell carcinoma, metastatic NOS
8071/3	Squamous cell carcinoma, keratinizing NOS
8072/3	Squamous cell carcinoma, large cell, nonkeratinizing
8073/3	Squamous cell carcinoma, small cell, nonkeratinizing
8074/3	Squamous cell carcinoma, spindle cell
8075/3	Adenoid squamous cell carcinoma
8076/3	Squamous cell carcinoma, microinvasive
8082/3	Lymphoepithelial carcinoma
8094/3	Basosquamous carcinoma
8120/3	Transitional cell carcinoma NOS
8130/3	Papillary transitional cell carcinoma
8140/3	Adenocarcinoma NOS
8140/6	Adenocarcinoma, metastatic NOS
8141/3	Scirrhous adenocarcinoma
8143/3	Superficial spreading adenocarcinoma
8144/3	Adenocarcinoma, intestinal type
8145/3	Carcinoma, diffuse type
8190/3	Trabecular adenocarcinoma
8200/3	Adenoid cystic carcinoma
8201/3	Cribriform carcinoma
8210/3	Adenocarcinoma in adenomatous polyp
8211/3	Tubular adenocarcinoma
8230/3	Solid carcinoma NOS
8231/3	Carcinoma simplex
8250/3	Bronchioloalveolar adenocarcinoma
8251/3	Alveolar adenocarcinoma
8260/3	Papillary adenocarcinoma NOS
8261/3	Adenocarcinoma in villous adenoma
8263/3	Adenocarcinoma in tubulovillous adenoma
8290/3	Oxyphilic adenocarcinoma
8310/3	Clear cell adenocarcinoma NOS
8323/3	Mixed cell adenocarcinoma
8330/3	Follicular adenocarcinoma NOS
8340/3	Papillary carcinoma, follicular variant
8380/3	Endometrioid carcinoma
8401/3	Apocrine adenocarcinoma
8410/3	Sebaceous adenocarcinoma
8420/3	Ceruminous adenocarcinoma
8430/3	Mucoepidermoid carcinoma
8440/3	Cystadenocarcinoma NOS
8441/3	Serous cystadenocarcinoma NOS
8442/3	Serous cystadenoma, borderline malignancy
8462/3	Papillary serous cystadenoma, borderline malignancy
8470/3	Mucinous cystadenocarcinoma NOS
8472/3	Mucinous cystadenoma, borderline malignancy
8480/3	Mucinous adenocarcinoma
8481/3	Mucin-producing adenocarcinoma
8490/3	Signet ring cell carcinoma
8490/6	Metastatic signet ring cell carcinoma
8500/3	Infiltrating duct carcinoma
8510/3	Medullary carcinoma NOS
8550/3	Acinar cell carcinoma
8560/3	Adenosquamous carcinoma
8562/3	Epithelial-myoepithelial carcinoma
8570/3	Adenocarcinoma with squamous metaplasia
8572/3	Adenocarcinoma with spindle cell metaplasia
8802/3	Giant cell sarcoma
8980/3	Carcinosarcoma NOS
**ICD-O-3**	**Term**
C34.0	Main bronchus
C34.1	Upper lobe, lung
C34.2	Middle lobe, lung (right lung only)
C34.3	Lower lobe, lung
C34.8	Overlapping lesion of lung
C34.9	Lung, NOS

## References

[1] Brenner D.R., Weir H.K., Demers A.A., Ellison L.F., Louzado C., Shaw A., Turner D., Woods R.R., Smith L.M. (2020). Projected estimates of cancer in Canada in 2020. Can. Med. Assoc. J..

[2] Ontario Health (Cancer Care Ontario) (2020). Ontario Cancer Statistics 2020.

[3] Temel J.S., Greer J.A., Muzikansky A., Gallagher E.R., Admane S., Jackson V.A., Dahlin C.M., Blinderman C.D., Jacobsen J., Pirl W.F. (2010). Early Palliative Care for Patients with Metastatic Non–Small-Cell Lung Cancer. N. Engl. J. Med..

[4] Wang Y., Van Dam A., Slaven M., Ellis K.J., Goffin J.R., Juergens R.A., Ellis P.M. (2019). Resource use in the last three months of life by lung cancer patients in southern Ontario. Curr. Oncol..

[5] Whitney R.L., Bell J.F., Tancredi D.J., Romano P.S., Bold R.J., Joseph J.G. (2017). Hospitalization Rates and Predictors of Rehospitalization among Individuals with Advanced Cancer in the Year after Diagnosis. J. Clin. Oncol. Off. J. Am. Soc. Clin. Oncol..

[6] Iyer S., Taylor-Stokes G., Roughley A. (2013). Symptom burden and quality of life in advanced non-small cell lung cancer patients in France and Germany. Lung Cancer.

[7] Farbicka P., Nowicki A. (2013). Palliative care in patients with lung cancer. Contemp. Oncol..

[8] Ramirez R.A., Lu J., Thomas K.E.H. (2018). Quality of life for non-small cell lung cancer patients in the age of immunotherapy. Transl. Lung Cancer Res..

[9] Henson L.A., Edmonds P., Johnston A., Johnson H.E., Ng Yin Ling C., Sklavounos A., Ellis-Smith C., Gao W. (2020). Population-Based Quality Indicators for End-of-Life Cancer Care: A Systematic Review. JAMA Oncol..

[10] Barbera L., Seow H., Sutradhar R., Chu A., Burge F., Fassbender K., McGrail K., Lawson B., Liu Y., Pataky R. (2015). Quality Indicators of End-of-Life Care in Patients with Cancer: What Rate Is Right?. J. Oncol. Pract..

[11] Barbera L., Seow H., Sutradhar R., Chu A., Burge F., Fassbender K., McGrail K., Lawson B., Liu Y., Pataky R. (2015). Quality of end-of-life cancer care in Canada: A retrospective four-province study using administrative health care data. Curr. Oncol..

[12] Bekelman J.E., Halpern S.D., Blankart C.R., Bynum J.P., Cohen J., Fowler R., Kaasa S., Kwietniewski L., Melberg H.O., Onwuteaka-Philipsen B. (2016). Comparison of Site of Death, Health Care Utilization, and Hospital Expenditures for Patients Dying with Cancer in 7 Developed Countries. JAMA J. Am. Med. Assoc..

[13] Cheung M.C., Earle C.C., Rangrej J., Ho T.H., Liu N., Barbera L., Saskin R., Porter J., Seung S.J., Mittmann N. (2015). Impact of aggressive management and palliative care on cancer costs in the final month of life. Cancer.

[14] Ho T.H., Barbera L., Saskin R., Lu H., Neville B.A., Earle C.C. (2011). Trends in the aggressiveness of end-of-life cancer care in the universal health care system of Ontario, Canada. J. Clin. Oncol. Off. J. Am. Soc. Clin. Oncol..

[15] Wang S.Y., Hall J., Pollack C.E., Adelson K., Bradley E.H., Long J.B., Gross C.P. (2016). Trends in end-of-life cancer care in the Medicare program. J. Geriatr. Oncol..

[16] Gonsalves W.I., Tashi T., Krishnamurthy J., Davies T., Ortman S., Thota R., Aldoss I., Ganta A., Kalaiah M., Didwaniya N. (2011). Effect of palliative care services on the aggressiveness of end-of-life care in the Veteran’s Affairs cancer population. J. Palliat. Med..

[17] Michael N., Beale G., O’Callaghan C., Melia A., DeSilva W., Costa D., Kissane D., Shapiro J., Hiscock R. (2019). Timing of palliative care referral and aggressive cancer care toward the end-of-life in pancreatic cancer: A retrospective, single-center observational study. BMC Palliat. Care.

[18] Scibetta C., Kerr K., McGuire J., Rabow M.W. (2016). The Costs of Waiting: Implications of the Timing of Palliative Care Consultation among a Cohort of Decedents at a Comprehensive Cancer Center. J. Palliat. Med..

[19] Jang R.W., Krzyzanowska M.K., Zimmermann C., Taback N., Alibhai S.M. (2015). Palliative care and the aggressiveness of end-of-life care in patients with advanced pancreatic cancer. J. Natl. Cancer Inst..

[20] World Health Organization WHO Definition of Palliative Care. http://www.who.int/cancer/palliative/definition/en/.

[21] Haun M.W., Estel S., Rucker G., Friederich H.C., Villalobos M., Thomas M., Hartmann M. (2017). Early palliative care for adults with advanced cancer. Cochrane Database Syst. Rev..

[22] Irwin K.E., Greer J.A., Khatib J., Temel J.S., Pirl W.F. (2013). Early palliative care and metastatic non-small cell lung cancer. Chronic Respir. Dis..

[23] Howie L., Peppercorn J. (2013). Early palliative care in cancer treatment: Rationale, evidence and clinical implications. Ther. Adv. Med. Oncol..

[24] Zimmermann C., Swami N., Krzyzanowska M., Hannon B., Leighl N., Oza A., Moore M., Rydall A., Rodin G., Tannock I. (2014). Early palliative care for patients with advanced cancer: A cluster-randomised controlled trial. Lancet.

[25] Yoong J., Park E.R., Greer J.A., Jackson V.A., Gallagher E.R., Pirl W.F., Back A.L., Temel J.S. (2013). Early palliative care in advanced lung cancer: A qualitative study. JAMA Intern. Med..

[26] Hui D., Kim S.H., Roquemore J., Dev R., Chisholm G., Bruera E. (2014). Impact of timing and setting of palliative care referral on quality of end-of-life care in cancer patients. Cancer.

[27] Canadian Cancer Society (2016). Right to Care: Palliative Care for All Canadians.

[28] Khandelwal N., Brumback L.C., Halpern S.D., Coe N.B., Brumback B., Curtis J.R. (2017). Evaluating the Economic Impact of Palliative and End-of-Life Care Interventions on Intensive Care Unit Utilization and Costs from the Hospital and Healthcare System Perspective. J. Palliat. Med..

[29] Pilkey J., Downar J., Dudgeon D., Herx L., Oneschuk D., Schroder C., Schulz V. (2017). Palliative Medicine—Becoming a Subspecialty of the Royal College of Physicians and Surgeons of Canada. J. Palliat. Care.

[30] Clarke E.A., Marrett L.D., Kreiger N. (1991). Cancer registration in Ontario: A computer approach. IARC Sci. Publ..

[31] Kralj B. (2008). Measuring Rurality—RIO2008 BASIC: Methodology and Results. http://www.eriestclairlhin.on.ca/Page.aspx?id=11606.

[32] Southern D.A., Quan H., Ghali W.A. (2004). Comparison of the Elixhauser and Charlson/Deyo Methods of Comorbidity Measurement in Administrative Data. Med. Care.

[33] Parr J.D., Zhang B., Nilsson M.E., Wright A., Balboni T., Duthie E., Paulk E., Prigerson H.G. (2010). The influence of age on the likelihood of receiving end-of-life care consistent with patient treatment preferences. J. Palliat. Med..

[34] Verleye L., De Gendt C., Vrijens F., Schillemans V., Camberlin C., Silversmit G., Stordeur S., Van Eycken E., Dubois C., Robays J. (2018). Patterns of care for non-small cell lung cancer patients in Belgium: A population-based study. Eur. J. Cancer Care.

[35] Philip J., Hudson P., Bostanci A., Street A., Horey D.E., Aranda S., Zordan R., Rumbold B.D., Moore G., Sundararajan V. (2015). Metastatic non-small cell lung cancer: A benchmark for quality end-of-life cancer care?. Med. J. Aust..

[36] Bylicki O., Rivière F., Tournier C., Canoui-Poitrine F., Grassin F., Margery J., Prodel M., Vainchtock A., Assié J.B., Chouaïd C. (2021). Factors Associated with Aggressiveness of End-of-Life Care for Lung Cancer Patients and Associated Costs of Care. Clin. Lung Cancer.

[37] Lehto R.H., Miller S.E.L., Flanigan M., Wyatt G. (2018). Mental health in patients with advanced cancer at the end of life: Evaluation of evidence and future directions. Expert Rev. Qual. Life Cancer Care.

[38] Park T., Hegadoren K., Workun B. (2020). Working at the Intersection of Palliative End-of-Life and Mental Health Care: Provider Perspectives. J. Palliat. Care.

[39] Rosenstein D.L. (2011). Depression and end-of-life care for patients with cancer. Dialogues Clin. Neurosci..

[40] Vodermaier A., Linden W., MacKenzie R., Greig D., Marshall C. (2011). Disease stage predicts post-diagnosis anxiety and depression only in some types of cancer. Br. J. Cancer.

[41] Lo C., Zimmermann C., Rydall A., Walsh A., Jones J.M., Moore M.J., Shepherd F.A., Gagliese L., Rodin G. (2010). Longitudinal study of depressive symptoms in patients with metastatic gastrointestinal and lung cancer. J. Clin. Oncol. Off. J. Am. Soc. Clin. Oncol..

[42] Kroschinsky F., Stölzel F., von Bonin S., Beutel G., Kochanek M., Kiehl M., Schellongowski P., on behalf of the Intensive Care in Hematological and Oncological Patients (iCHOP) Collaborative Group (2017). New drugs, new toxicities: Severe side effects of modern targeted and immunotherapy of cancer and their management. Crit. Care.

[43] Louie A.V., Li L., Jenkyn K.B., Allen B., Rodrigues G.B., Warner A., Palma D.A., Shariff S.Z. (2018). A population-based analysis of outcomes after radiotherapy in intensive care unit patients with lung cancer. J. Thorac. Dis..

[44] Shokar S., Buldo E., Siu L.L., Hansen A.R., Spreafico A., Doi J., Carlsson L., Bedard P.L. (2018). Patient knowledge, attitudes, and expectations of cancer immunotherapies. J. Clin. Oncol..

[45] Sharma R.K., Prigerson H.G., Penedo F.J., Maciejewski P.K. (2015). Male-female patient differences in the association between end-of-life discussions and receipt of intensive care near death. Cancer.

[46] Earle C.C., Landrum M.B., Souza J.M., Neville B.A., Weeks J.C., Ayanian J.Z. (2008). Aggressiveness of cancer care near the end of life: Is it a quality-of-care issue?. J. Clin. Oncol. Off. J. Am. Soc. Clin. Oncol..

[47] Hales S., Chiu A., Husain A., Braun M., Rydall A., Gagliese L., Zimmermann C., Rodin G. (2014). The Quality of Dying and Death in Cancer and Its Relationship to Palliative Care and Place of Death. J. Pain Symptom Manag..

[48] Monier P.A., Chrusciel J., Ecarnot F., Bruera E., Sanchez S., Barbaret C. (2020). Duration of palliative care involvement and cancer care aggressiveness near the end of life. BMJ Support. Palliat. Care.

[49] Colombet I., Bouleuc C., Piolot A., Vilfaillot A., Jaulmes H., Voisin-Saltiel S., Goldwasser F., Vinant P. (2019). Multicentre analysis of intensity of care at the end-of-life in patients with advanced cancer, combining health administrative data with hospital records: Variations in practice call for routine quality evaluation. BMC Palliat. Care.

